# Earwax cortisol in bovines: biological and seasonal influences

**DOI:** 10.2478/jvetres-2026-0054

**Published:** 2026-10-14

**Authors:** Mohammad Ataallahi, Geun-Woo Park, Dong-Woo Kim, Kyu-Hyun Park

**Affiliations:** Department of Animal Industry Convergence, College of Animal Life Sciences, Kangwon National University, Chuncheon 24341, Korea

**Keywords:** cattle, cerumen cortisol, chronic stress, non-invasive sampling

## Abstract

**Introduction:**

Accurate stress assessment in animals requires reliable biomarkers, yet the impact of intrinsic (sex and age) and extrinsic (seasonal) factors on earwax cortisol concentrations (ECCs) has not been thoroughly studied. Therefore, the effects of sex, age and seasonal climate on ECC were investigated in Hanwoo cattle (*Bos taurus coreanae*).

**Material and Methods:**

Monthly earwax samples were collected from the left ear canal of 22 individual cattle. These comprised 7 males and 15 females as a cohort of 19 animals in March 2022 with 3 additional calves recruited through the study period up to August 2022. Their overall mean age was 32.7 ± 19.6 months. Earwax cortisol was extracted using methanol and quantified by a validated enzyme immunoassay. Based on the temperature-humidity index (THI), conditions were classified as cold stress (THI < 38), no stress (THI 38–72) or heat stress (THI > 72). Months were grouped into cool and warm seasons.

**Results:**

Earwax cortisol concentrations were higher in females than males (30.3 *vs* 20.6 pg/mg; P-value = 0.0015) and differed by THI category (P-value = 0.0074), peaking in females when the THI was 77 (July). Females older than 25 months had higher ECC than age-matched males (31.4 *vs* 15.4 pg/mg; P-value = 0.0036). Season alone did not affect ECC (P-value = 0.4943), although females maintained higher ECCs than males in the cool season (P-value = 0.0350). In females, the ECC was positively associated with age (β = 0.113 pg/mg/month by linear regression; P-value = 0.0121), but not in males.

**Conclusion:**

Sex is the primary influence on ECC, age and environmental conditions being lesser ones. Both intrinsic biological and extrinsic environmental factors should be considered when interpreting chronic stress in bovines.

## Introduction

In clinical veterinary studies, cortisol is widely used as an indicator of physiological stress and animal welfare status. Various environmental and physiological challenges can activate the hypothalamic–pituitary–adrenal (HPA) axis, which leads to the sequential release of corticotropin-releasing hormone from the hypothalamus, adrenocorticotropic hormone from the pituitary gland, and finally cortisol from the adrenal cortex (2). Cortisol, as a lipid-soluble hormone, can easily diffuse through cell membranes and exerts negative feedback on both the hypothalamus and pituitary to regulate its basal blood concentrations (2). The body matrices used for cortisol measurement differ in their properties and analytical limitations, which complicates the evaluation of chronic stress in animals. Conventional fluid matrices such as blood, saliva and urine are widely used in studies of animal stress physiology (2). These matrices reflect short-term activity of the HPA axis and have been applied to evaluate physiological responses to various stressors, such as environmental challenges, management procedures and disease conditions. Nevertheless, fluid matrices often require repeated sampling because cortisol concentrations fluctuate with circadian rhythms. In addition, sample collection can be invasive and may itself induce stress, and it typically requires trained personnel, specialised equipment and appropriate storage conditions. Cortisol measurement may also be influenced by interactions with endogenous components or sample degradation. Thus, non-fluid matrices such as hair, feather, wool, nails, scales and teeth, which archive hormone deposition over extended periods (weeks to months), have been increasingly used to evaluate chronic stress (2).

Recently, cerumen (earwax) has been investigated as a potential matrix for chronic cortisol monitoring (15, 30). Because the lipid-rich matrix of cerumen accretes gradually in the ear canal, cortisol incorporated during its formation is preserved within it, yielding a time-integrated record of physiological stress over weeks to months (4, 6). This characteristic allows investigators to evaluate historical patterns of cortisol exposure longitudinally (4, 6). Although evidence from humans and animal studies supports the use of earwax for long-term endocrine monitoring, its application in livestock stress has only recently been explored. For example, Stonecipher *et al*. (28) used earwax to determine cattle exposure to teratogenic lupine, while Lee *et al*. (19) used it to determine sheep exposure to death camas (*Zigadenus paniculatus*). In previous studies (4, 6), earwax was highlighted as a potential biomatrix for cortisol evaluation in cattle and the need was emphasised for comprehensive investigation. To validate earwax cortisol concentration (ECC) as a biomarker of chronic stress in cattle, biological factors such as sex, age, breed and reproductive or lactational status and environmental factors such as season, temperature and humidity, housing conditions and management practices should be considered. This is because cortisol secretion is influenced by these variables (20), and therefore, they may confound interpretation and limit the utility of earwax.

Hanwoo cattle (*Bos taurus coreanae*), Korea’s native beef breed (21), provide an ideal model for investigating cortisol in relation to intrinsic and extrinsic factors. Because pastureland is limited, Hanwoo are raised almost exclusively in indoor housing systems, which allow controlled feeding and watering, but also expose animals to stressors such as mechanical noise and restricted movement, while also not fully mitigating seasonal climate extremes which are further stressors (3, 21). Weather-induced stress is particularly relevant for cortisol assessment. We hypothesised that both natural biological characteristics and environmental conditions would influence ECC. Therefore, we investigated whether ECC is influenced by sex (male *vs* female), age group (calves, young cattle, and adult cattle), and season (March–August, covering cool to warm months) in Hanwoo cattle.

## Material and Methods

### Experimental animals

This study was carried out on the livestock research and commercial farm at Kangwon National University, Chuncheon, Korea (37°56′24.1″N, 127°46′57.1″E). Experimental cattle were housed in a 1,071 m^2^ steel-frame winch-curtain barn with approximately 10-cm-deep sawdust bedding. Animals were kept in pens containing one to four individuals and maintained under the same environmental conditions. All cattle received a standardised diet of roughage, pellets and concentrates twice daily (09:00 and 17:00), with *ad libitum* access to fresh water (4, 6).

A total of 22 individual cattle contributed data over the study period, March–August 2022, with repeated sampling from the same animals. The cohort comprised 7 males and 15 females with an overall mean age of 32.7 ± 19.6 months, the males being at 20.3 ± 8.4 months and ranging between 4 and 34 and the females being at 37.8 ± 20.9 months and ranging between 4 and 104. The study began with 19 animals (6 males and 13 females) in March, but there was recruitment and attrition during the study: 1 male calf and 1 female calf were added in May, 1 female calf was added in June and 1 male calf was removed in August (Table 1). The number of earwax samples collected per month was 19 in March, 18 in April, 17 in May, 18 in June, 17 in July and 12 in August (Table 1), yielding a total of 101 through the study period. A small number of individual cortisol measurements failed because of assay or technical errors (Table 1); these losses were not attributable to a specific animal and are reported as their combined effect on monthly sample size.

**Table 1.** Summary of Korean native cattle examined between March and August 2022

Month	Male	Female	Average age in months	Male calves aged <12	Female calves aged <12	Young females 12–25	Young males 12–25	Adult females aged >25	Adult males aged >25	Total
March	6	13	34 ± 24	2	1	3	3	9	1	19
April	6	12	31 ± 17	2	1	3	2	8	2	18
May	5	12	32 ± 18	1*	1*	2	2	9	2	17
June	5	13	32 ± 19	1	2**	1	2	10	2	18
July	4	13	33 ± 19	1	2	1	2	10	1	17
August	3	9	35 ± 21	0***	2	0	2	7†	1	12
Total individual animals	7	15††	-	3	3	3††	3††	11††	2††	-
Total observations	29	72	-	7	9	10	13	53	9	-

*: new 4-month-old male and female added in May;

** – one 5-month-old female added in June;

*** – one 7-month-old male removed in August;

† – missed sampling from a few adult females;

†† – because animals aged into a higher category during the study, 3 animals are counted in two category columns each (one male moved from young cattle group to adult cattle group; two females moved from the young-cattle group to the adult-cattle group).

Total observations – monthly sample count

At each sampling time point, animals were retrospectively classified into three age groups: calves (<12 months), young cattle (12–25 months), and adult cattle (>25 months), to examine age-related differences. Because animals aged into higher categories during the study, some individuals were represented in more than one age category across the six months (Table 1). Across the full study, the calf group included 3 males and 3 females, the young-cattle group included 3 males and 3 females, and the adult-cattle group included 2 males and 11 females. Because 1 male and 2 females crossed an age-group boundary during the study, they were counted in two categories. After accounting for this overlap, the study included 22 unique animals: seven males and 15 females. The marked imbalance in the >25-month category (2 males *vs* 11 females) reflects a structural feature of the source farm rather than a sampling choice: most males reared on the farm are castrated and sold or slaughtered relatively young, while females are retained longer for breeding, which limited the precision of sex comparisons within that specific age category and is addressed further in the Discussion.

### Earwax collection

To ensure that only newly secreted earwax was collected, the inner surface of the external ear canal was gently wiped with tissue (Kimwipe; Kimberly-Clark, Irving, TX, USA) moistened with 70% ethanol approximately one month prior to sampling. This step removed older deposits and facilitated the collection of fresh earwax at the subsequent sampling. Care was taken not to insert the wipe deeply into the ear canal to avoid causing a blockage or any risk of tympanic membrane injury (28). Earwax samples were collected monthly in the morning (7:00–9:00 am) to minimise circadian variation. Cattle were restrained in headlocks for safe handling, and earwax was gently obtained from the left ear using a sterile curette (4, 6). After collection, the ear canal was again wiped with a Kimwipe moistened with ethanol to reset deposition, such that subsequent samples reflected approximately one month of accumulation. Because the bovine ear canal is narrow and lies close to the eardrum, all procedures were performed with caution to avoid injury or disturbance to hearing. All samples were stored in 1.5 mL Eppendorf tubes at −21°C until laboratory analysis.

### Earwax cortisol extraction

A single extraction procedure was performed using methyl alcohol. The extract was then evaporated and reconstituted with sample diluent (6). From each earwax sample (>500 mg), a 50 mg subsample was weighed into a 1.5-mL microcentrifuge tube. To each tube, 1.0 mL of extra-pure methyl alcohol (99.5%; Daejung Chemicals & Metals, Gyeonggi-do, Korea) was added, and the tubes were sealed with parafilm. Samples were vortexed for 1 min to homogenise the earwax samples with the methyl alcohol, then they were continuously shaken at 40 rpm for 24 h using a tube rotator (MX-RL-Pro; Scilogex, Rocky Hill, CT, USA) at room temperature (22–24°C) (4). After incubation, the samples were then centrifuged at 14,000 rpm for 1 min using a refrigerated microcentrifuge (1730R; Gyrozen, Seoul, South Korea). The resulting supernatants (0.6 mL) were carefully transferred to clean microtubes and evaporated in an incubator at 38°C. The dried residues were stored at −21°C until analysis.

### Earwax cortisol quantification

The extracted earwax samples were thawed at room temperature, after which 0.4 mL of the assay buffer was added and the sample thoroughly vortexed. They were centrifuged at 1,500 × *g* for 15 min in a refrigerated microcentrifuge prior to cortisol quantification using a commercial cortisol ELISA kit (ADI-900-071; Enzo Life Sciences, Farmingdale, NY, USA), previously validated for earwax cortisol measurement (4, 6). The kit is designed for diverse biomatrices and has a detection range of 156–10,000 pg/mL and a sensitivity of 56.72 pg/mL. Each sample (0.1 mL) was run in duplicate in a 96 well-plate per the manufacturer’s protocol to improve assay accuracy and reliability. The optical density of the samples was read at 405 nm using a microplate reader (SpectraMax; Molecular Device, San Jose, CA, USA), and cortisol concentrations were calculated using a four-parameter logistic calibration curve fitting. Final outputs were expressed as pg of cortisol per mg of earwax (3). To ensure assay reliability, the coefficient of variation for ECC was 5.6% intra-assay and 13.6% inter-assay, both within the accepted limits of ≤15% and ≤20%, respectively.

### Environmental variable measurements

Daily average, minimum and maximum air temperature and relative humidity were obtained from the Korea meteorological administration (KMA) website, (http://www.kma.go.kr/eng/index.jsp) during the experimental period. The Chuncheon KMA station (37°56ʹ50.1ʺN, 127°45ʹ17.5ʺE) is located in Gangwon-do, Korea, where the summers are hot, the winters are cold and the mean annual temperature is 11.1°C (24.6°C in August and −4.6°C in January) (5). The KMA is located about 2.5 km north of the experimental Hanwoo farms (5). We applied KMA data in the current study because no significant differences pertained to the environmental data between the Hanwoo farm and the KMA station (5). The temperature-humidity index (THI) is a widely accepted indicator used to quantify the heat stress experienced by bovines, as it reflects the combined effects of temperature and humidity (14). The THI calculation method has been previously described in detail (5). Table 2 presents the THI values indicative of environmental stressors in this study. A higher THI indicates a higher risk of heat stress. For beef cattle, heat stress has been classified as follows: no stress (38 < THI ≤ 72, all breeds), mild stress (72 < THI ≤ 77, in all breeds), moderate stress (77 < THI ≤ 86, in *Bos taurus*), severe stress (86 < THI ≤ 98, in all breeds) and dangerous stress (THI > 98, in all breeds) (18). Cold stress thresholds have been defined as follows: no stress (THI > 38), mild stress (25 < THI ≤ 38), moderate stress (8 < THI ≤ 25), high stress (−12 < THI ≤ 8), extreme stress (−25 < THI ≤ −12), and dangerous stress (THI ≤ −25) (14). The monthly earwax sampling from March to August captured the gradual transition from cooler to hotter months, which is typical of the Korean climate, and more frequent earwax sampling was unnecessary because cortisol is gradually deposited in earwax and the limited monthly production of this hormone makes repeated short-interval sampling impractical.

**Table 2.** Measured climate parameters in Chuncheon, South Korea where Korean native cattle earwax was sampled from March to August 2022

Month	Avg temp (°C)	Min temp (°C)	Max temp (°C)	Min humidity (RH%)	Avg humidity (RH%)	Avg THI	Max THI
March	5.7 ± 3.0	−0.9 ± 4.4	12.5 ± 3.5	36.5 ± 19.5	66.4 ± 14.8	45 ± 4^*^	56 ± 4
April	13.0 ± 3.7	5.1 ± 4.5	21.2 ± 4.5	26.0 ± 13.3	55.5 ± 11.7	56 ± 5^*^	65 ± 5
May	17.2 ± 2.9	9.4 ± 3.7	25.0 ± 3.3	28.6 ± 10.8	60.1 ± 7.4	62 ± 4^*^	70 ± 4
June	21.9 ± 3.0	17.3 ± 4.1	27.0 ± 3.6	54.8 ± 18.6	78.3 ± 13.1	70 ± 5^**^	75 ± 5
July	26.0 ± 1.6	22.2 ± 1.4	31.1 ± 2.9	60.1 ± 12.2	84.6 ± 5.6	77 ± 2^**^	81 ± 3
August	24.5 ± 2.7	21.4 ± 3.2	28.8 ± 3.4	67.1 ± 14.7	87.7 ± 8.2	74 ± 7^**^	79 ± 5

THI = (1.8 × temperature + 32) – ((0.55 – 0.0055 × relative humidity) × (1.8 × temperature – 26)) (5, 18); Avg THI – average temperature, average relative humidity; Max THI – maximum temperature, minimum relative humidity;

* – no stress;

** – mild heat stress

Statistical analyses were performed using SAS v. 9.4 (SAS Institute, Cary, NC, USA). The distribution of ECC was assessed using PROC UNIVARIATE; raw ECC values deviated significantly from normality (Shapiro–Wilk W = 0.974, P-value = 0.0008, n = 202). Effects of sex, age group, season and THI on ECC were evaluated using linear mixed models (PROC MIXED) fitted to cow-month mean ECC values (n = 101 observations from 22 cows), with cow as the subject and month as the repeated factor. An autoregressive (AR(1)) covariance structure was used, and degrees of freedom were estimated by the Kenward–Roger method. Because season was defined directly by THI range (the cool season in the THI range 45–62 and the warm season in 70–77), season and THI were not fitted in the same model; each was included separately with sex and age group as covariates. Four mixed models were fitted, corresponding to Figs 1–4: (1) sex and age group with THI as a continuous covariate (Fig. 1); (2) sex and age group with THI as a class variable and a sex × THI interaction (Fig. 2); (3) sex and age group with season and a sex × season interaction (Fig. 3); and (4) sex and age group with THI as a continuous covariate and a sex × age group interaction (Fig. 4). Pairwise comparisons of least-squares means were adjusted by the Tukey–Kramer method. Separately, simple linear regression (PROC REG) was used to examine the relationship between ECC and continuous age, overall and separately by sex, using all ELISA measurements (n = 202 from 101 samples) (Fig. 5). Statistical significance was set at P-value < 0.05.

**Fig. 1.**
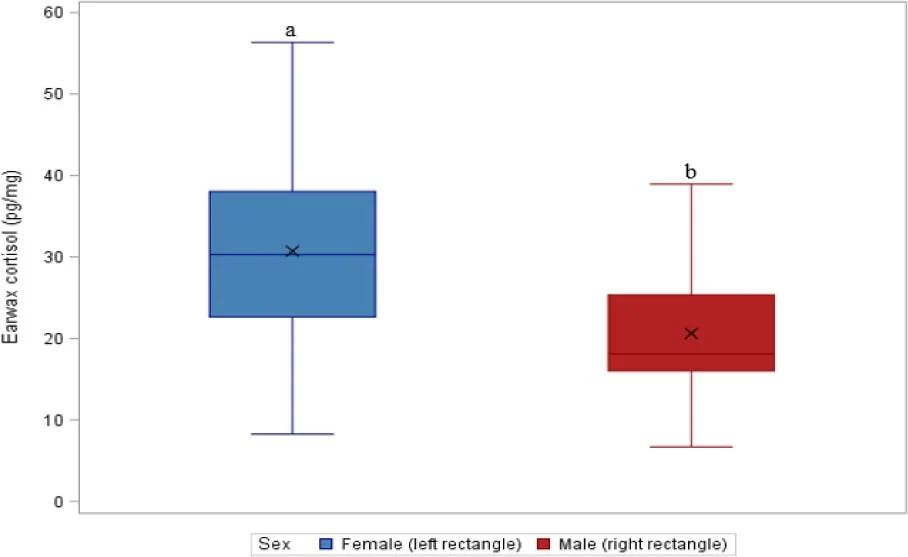
Boxplot of earwax cortisol concentration by sex in Hanwoo cattle. The horizontal lines within boxes indicate the median, the “×” marks indicate the mean, and whiskers indicate the minimum and maximum values. Different letters indicate a significant difference between sexes (Tukey–Kramer adjusted P-value < 0.05)

**Fig. 2.**
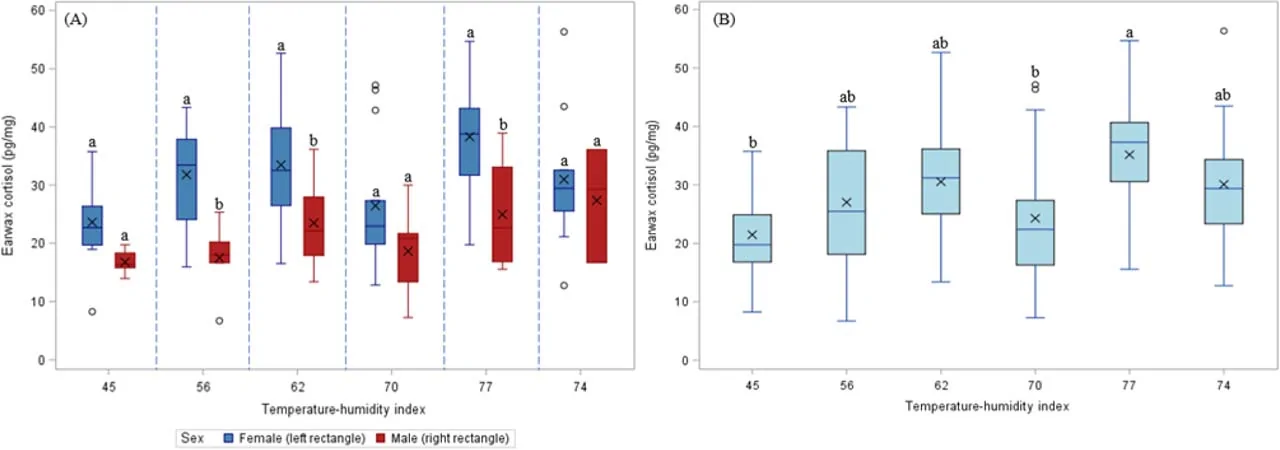
Earwax cortisol concentration (ECC) across temperature-humidity index levels corresponding to monthly conditions (March = 45, April = 56, May = 62, June = 70, July = 77 and August = 74). (A) Sex-specific ECC at each THI level. (B) ECC averaged for both sexes. Boxes represent the interquartile range, the horizontal lines within boxes indicates the median, the “×” marks indicate the mean, whiskers indicate the minimum and maximum values, and circles represent outliers. Shared letters indicate no significant difference. Different letters indicate significant differences (Tukey–Kramer adjusted P-value < 0.05)

**Fig. 3.**
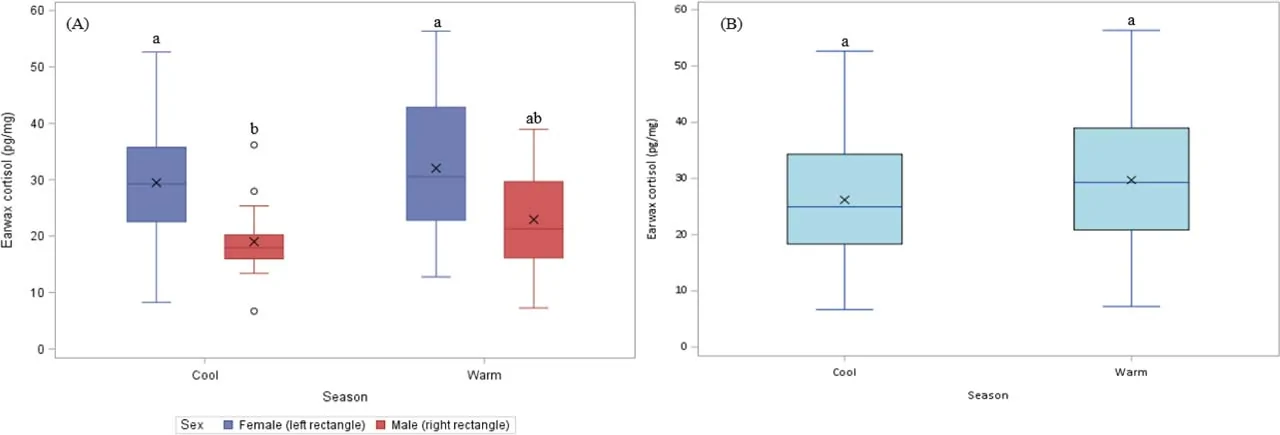
Earwax cortisol concentration by sex and season (cool March–May, THI 45–62; warm June–August, THI 70–77). (A) ECC by sex within each season. (B) ECC averaged for both sexes by season. Boxes represent the interquartile range, the horizontal lines within boxes indicate the median, the “×” marks indicate the mean, whiskers indicate the minimum and maximum values, and circles represent outliers. Different letters indicate significant differences (Tukey-Kramer adjusted P-value < 0.05)

**Fig. 4.**
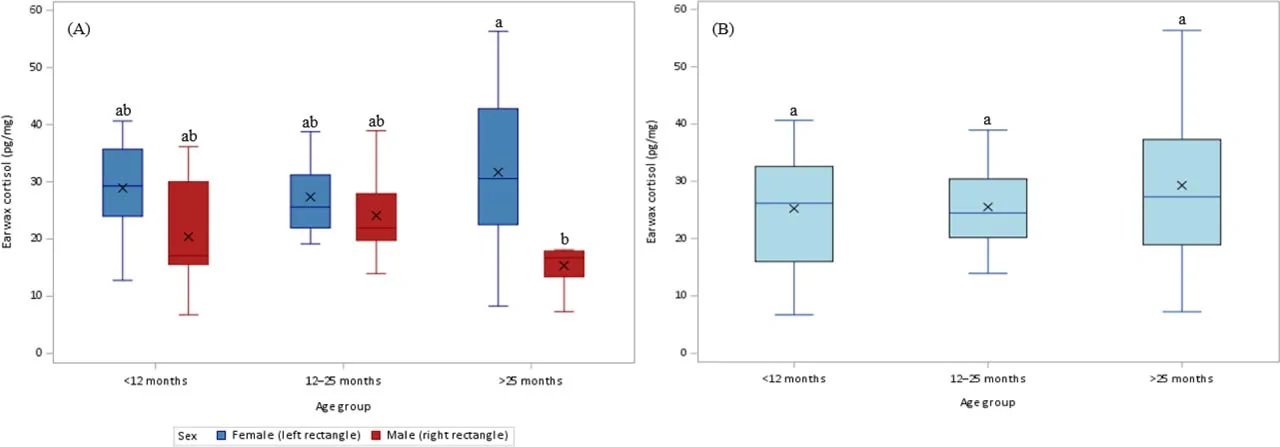
Earwax cortisol concentration (ECC) by sex and age group (<12, 12–25 and >25 months), adjusted for temperature-humidity index. (A) Sex-specific ECC by age group. (B) ECC by age group averaged for both sexes. Boxes represent the interquartile range, the horizontal line indicates the median, the “×” marks within boxes indicate the mean, and whiskers indicate the minimum and maximum values. Different letters indicate significant differences (Tukey-Kramer adjusted P-value < 0.05)

**Fig. 5.**
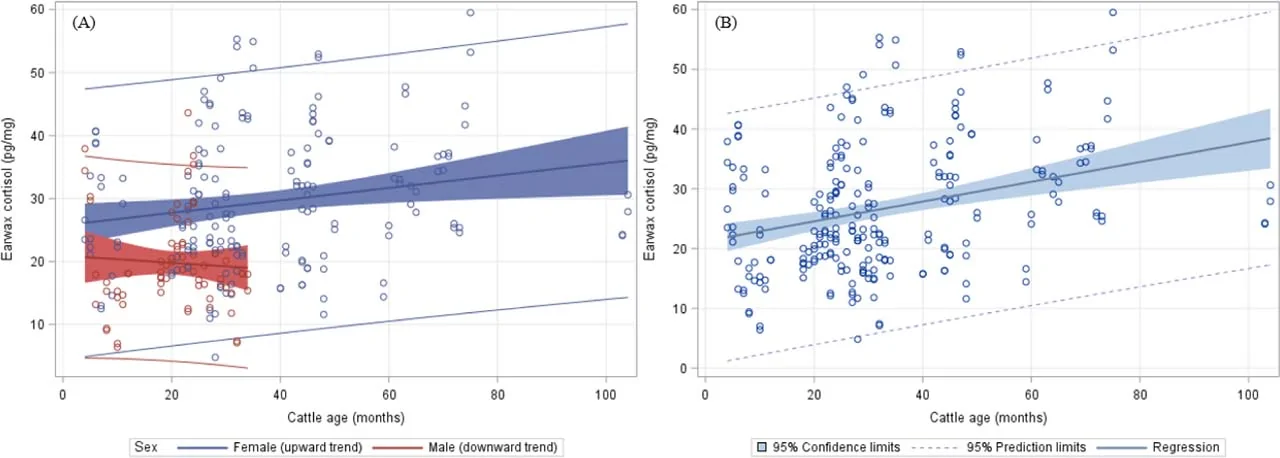
Relationship between cattle age and earwax cortisol concentration. (A) Sex-specific linear regressions based on individual ELISA measurements from females (144 measurements from 15 cattle) and males (58 measurements from 7 cattle). (B) Linear regression using the combined dataset (202 ELISA measurements from 101 biological earwax samples collected from 22 cattle). The solid lines represent the fitted regression, the shaded area denotes the 95% confidence interval, and the dashed lines indicate the 95% prediction interval

## Results

### Cortisol variation by sex and season

Sex had a significant effect on ECC (F_1,31.4_ = 12.08, P-value = 0.0015). Least-squares means showed that ECC was significantly higher in females (30.28 ± 1.72) than in males (20.60 ± 2.20), a difference of 9.68 ± 2.79 (95% confidence interval 4.00–15.36; t_31.4_ = 3.48, P-value = 0.0015, Tukey–Kramer adjusted) (Fig. 1).

Sex (F_1,28.1_ = 10.44, P-value = 0.0031) and THI (F_5,70.5_ = 3.46, P-value = 0.0074) each had a significant effect on ECC, whereas the sex × THI interaction was not significant (F_5,70_ = 0.64, P-value = 0.6687), indicating that the effect of THI on ECC did not differ significantly between sexes. Females showed consistently higher ECC than males across all THI levels (Fig. 2A). *Post-hoc* comparisons at each THI level showed that the sex difference was statistically significant at THI = 56, 62 and 77 (P-value = 0.0096, 0.0430 and 0.0147, respectively), but not at THI = 45, 70 or 74 (P-value > 0.10). Averaged for both sexes, the ECC increased progressively with rising THI, from a least-squares mean of 19.70 ± 2.48 pg/mg at THI = 45 to 31.05 ± 2.74 pg/mg at THI = 77 (F_5,70.5_ = 3.46, P-value = 0.0074). The ECC at THI = 77 was significantly higher than at THI = 45 (P-value = 0.0282) and THI = 70 (P-value = 0.0450); no other *post-hoc* comparisons among THI levels reached significance (Fig. 2B).

Sex had a significant effect on ECC (F_1,26.9_ = 10.81, P-value = 0.0028), whereas season (F_1,71.1_ = 0.47, P-value = 0.4943) and the sex × season interaction (F_1,72.4_ = 0.01, P-value = 0.9080) were not significant. Females showed consistently higher ECC than males, significantly so in the cool season (29.48 ± 2.21 *vs* 19.52 ± 2.85 pg/mg; F_1,37.6_ = 7.68, P-value = 0.0350), but not in the warm season (30.90 ± 2.30 *vs* 21.52 ± 3.41 pg/mg; F_1,42.1_ = 5.20, P-value = 0.1119) (Fig. 3A). Within each sex, ECC did not differ significantly between cool and warm seasons (females: P-value = 0.6006; males: P-value = 0.6352). When averaged for both sexes, the least-squares mean ECC did not differ significantly between the cool (24.50 ± 1.81 pg/mg) and warm (26.21 ± 2.06 pg/mg) seasons (F_1,71.1_ = 0.47, P-value = 0.4943) (Fig. 3B).

### Cortisol variation by sex and age group

Sex had a significant effect on ECC (F_1,33.8_ = 9.56, P-value = 0.0040), whereas age group (F_2,42_ = 0.69, P-value = 0.5078) and the sex × age group interaction (F_2,43.2_ = 2.18, P-value = 0.1257) were not significant. Females showed a higher ECC than males in all three age groups (Fig. 4A); however, this difference reached significance only in the >25-month-old age group (31.42 ± 1.55 *vs* 15.36 ± 3.75 pg/mg; t = 3.95, P-value = 0.0036), and was not significant in the <12-month-old (26.58 ± 3.90 *vs* 21.03 ± 4.32 pg/mg; P-value = 0.3468) or 12–25-month-old (29.06 ± 3.36 *vs* 24.53 ± 2.98 pg/mg; P = 0.3160) age groups. When averaged for both sexes, the least-squares mean ECC did not differ significantly among age groups (age group <12: 23.80 ± 2.90; age group 12–25: 26.80 ± 2.27; age group >25: 23.39 ± 2.03 pg/mg; F_2,42_ = 0.69, P-value = 0.5078) (Fig. 4B). No comparison between individual age groups reached significance (all P-value > 0.25).

Regression using the complete ELISA dataset (202 measurements, 101 samples and 22 cattle) showed in females that age was a significant positive predictor (β = 0.113 pg/mg/month, P-value = 0.0121); in males, the relationship was not significant (β = −0.160 pg/mg/month, P-value = 0.2050) (Fig. 5A). Pooled regression across all cattle confirmed a significant positive effect of age on ECC (F1, 200 = 19.59, P-value < 0.0001), with cortisol increasing by 0.174 pg/mg per month (R^2^ = 0.089) (Fig. 5B).

## Discussion

### Cortisol variation by sex and season

Ceruminous glands are specialised sweat glands located subcutaneously in the external ear canal and are modified apocrine glands that produce earwax together with sebaceous glands (24). The bovine external auditory canal is generally 0.6 cm in diameter and 10–13 cm in length (22). Earwax maintains eardrum flexibility while lubricating, waterproofing and cleansing the external auditory canal (22). In addition to its antibacterial properties (29) that protect the ear canal, earwax contains a diverse range of biomolecules such as lipids, proteins and metabolites making it a suitable matrix for retrospective biochemical analyses in cattle (10, 25, 27). Beyond its protective function, earwax has recently gained attention as a diagnostic matrix (12, 19, 28). It is less exposed to external factors and remains more stable over time and it offers a more controlled matrix for analysis, reducing variability and contamination risks (12).

This study is the first to our knowledge to examine ECC in cattle in relation to sex, age and seasonal variation. The results demonstrated clear sex-related differences, with females having higher mean cortisol levels and greater variability and yielding more frequent outliers than males. These findings are consistent with the review by Russell *et al*. (26), who noted the influence of age, sex and season on hair cortisol concentrations across species. Biriukova *et al*. (8), reported that heifers had higher and more widely varying mean blood cortisol levels in response to stressors than bulls, indicating a more variable stress response in females. In blood, only 5–10% of cortisol exists in its unbound (biologically active) form, while the remainder is bound to serum proteins (1). Unbound cortisol enters biomatrices through intracellular mechanisms (1). Female cattle may exhibit greater endocrine responsiveness owing to multiple factors, such as hormonal cycles, reproductive physiology or sex-specific behavioural stress responses (17). Female cattle, especially those in production (lactating or pregnant), can experience higher chronic stress than males of the same age because of the greater metabolic and physiological demands associated with reproductive and milk production activities. While males often react more to acute stressors related to handling or breeding, females in production conditions may be subjected to continuous stress from repeated calving, lactation and associated nutritional and environmental challenges (17). The observation that extreme values (>50 pg/mg) occurred predominantly in females suggests that ECCs may vary individually more in females, particularly under challenging physiological or environmental conditions. Supporting this interpretation, dams appeared more sensitive to environmental stressors and showed signs of chronic stress, which reduced feed intake and body condition scores; this creates a negative energetic balance that can impair multiple physiological processes, including reproductive performance (13).

Sex had a significant effect on ECC, whereas season showed no overall effect, although the concentrations in males differed from those in females in each season. Uetake *et al.* (31) reported the cortisol level in the hair of lactating cows changed by season in combination with region. Comparable findings have been reported in humans, where earwax production and composition show seasonal variation, with triglyceride content decreasing from winter to summer, while sex played only a minor role (9). This suggests that seasonal climate variation may alter earwax physiology across species, even if the extent of sex effects differs. Although cortisol concentrations’ tendency to be higher during the warm season was not statistically significant when averaged for both sexes, the trend was consistent with cattle experiencing greater heat stress as indicated by higher THI values. These findings align with previous research showing that warm environmental conditions increase circulating cortisol in cattle (23). Importantly, females showed higher ECC than males in both seasons, although this difference was statistically significant only in the cool season; the sex × season interaction was not significant, indicating no strong evidence that the seasonal environment altered the sex effect. This highlights the importance of accounting for both biological and environmental covariates when interpreting ECC as an indicator of stress. Monthly variations in ECC reflected THI-dependent trends, with the lowest ECC recorded in March (THI = 45). According to published thresholds, a higher THI indicates a greater risk of heat stress (18), and cattle exposed to THIs between 38 and 70 are considered to be under no environmental stress. These non-stressful conditions likely explain the low ECCs observed in March. In contrast, the highest ECC was observed in July (THI 77), when the THI was classified as causing mild heat stress. This finding is consistent with the role of thermal stress in activating the adrenal axis and reflecting heightened HPA axis activity as cattle adjust metabolic and endocrine pathways to maintain thermal balance (18).

### Cortisol variation by sex and age

According to Dantzer *et al*. (11), sex, reproductive status and age of animals all have the ability to influence adrenal glucocorticoid production. For instance, findings in female red squirrels in late pregnancy showed significantly higher faecal glucocorticoid metabolite levels than non-reproductive females, whereas male levels remained relatively stable. Thus, reproductive condition may interact with age to amplify stress-hormone deposition in females. Large variations between individuals must also be considered.

Among females specifically, cattle older than 25 months had the highest mean ECC, although age-group differences were not statistically significant overall. This is consistent with earlier findings in calves, where significant age-related differences in cortisol dynamics were reported, indicating that cortisol regulation and stress responsiveness vary across developmental stages (8). This trend in females may suggest a cumulative effect of chronic environmental or physiological stressors over time (7), although this within-sex pattern did not reach statistical significance. However, our previous work on Hanwoo hair cortisol showed the opposite trend, where neonatal calves had higher cortisol concentrations than heifers and dams, likely reflecting the physiological instability of early life (3). In dairy cattle, hair samples from two-year-old cows were found to contain lower cortisol concentrations than those obtained from 15-day-old calves (7). A differential pattern between sexes was noted with increasing age, although the sex and age group interaction did not reach statistical significance. Females in the group older than 25 months exhibited the highest concentrations and variability, whereas males showed comparatively stable and lower values across all age groups. However, the >25-month male group included only two animals, because the study herd’s structure reflected typical commercial management practices in which most males are removed from the herd at a young age; this small sample size limited the precision of this estimate and warrants cautious interpretation. Interestingly, Cipriani *et al*. (9) reported no sex-related differences in earwax composition in humans, supporting the idea that sex hormones may only play a minor role in earwax production. In contrast, our results in cattle suggest that sex-related endocrine physiology and reproductive activity exert a stronger influence on ECC, particularly in older females. This outcome may reflect reproductive activity in older females, including gestation or lactation, which may elevate cortisol. Given this limitation, our primary evidence for an age-related effect on ECC comes from the continuous-age regression (Fig. 5), which incorporates all individuals without partitioning them into small discrete age-by-sex categories and is therefore less sensitive to the sex/age-group imbalance noted above. Regression analysis showed that age was a modest predictor of ECC, with cortisol levels increasing with age. This is consistent with what was reported by Baier *et al*. (7): that higher hair cortisol concentrations occur in older steers. Whereas hair cortisol primarily reflects circulating blood cortisol during hair growth, the biological origin of earwax cortisol remains less well defined and may involve contributions from glandular secretions in the ear canal. This difference in source could partly explain why age-related patterns in hair and earwax cortisol do not always align, and highlights the need for caution when comparing across biomatrices.

### Implications of earwax cortisol as a biomarker

Frequent or excessive collection may compromise the protective role of earwax, potentially disrupting ear canal physiology and increasing the risk of irritation and infection (27). Because these risks have not been studied in livestock, careful sampling protocols are necessary to protect animal welfare. A key advantage of earwax is its slow and cumulative secretion, which is largely unaffected by circadian rhythms and can therefore act as a biological archive of chronic stress exposure. Unlike blood or saliva, which require repeated sampling and may themselves induce stress, earwax collection is minimally invasive and less disruptive to the animal’s physiological status (19, 28). However, factors such as sex, age and season influence sebaceous and ceruminous gland activity, thereby affecting earwax quantity. Therefore, variation in cortisol levels may partly reflect glandular activity rather than systemic HPA-axis function. Despite its potential, research on earwax remains limited compared with investigations of more established biomatrices such as blood, saliva or hair. Standardised protocols for collection, extraction and interpretation are still lacking because too few studies have addressed the inner ear canal anatomy of bovines. Practical challenges also persist, such as cattle being highly sensitive around the head and ears and often making sudden movements during handling. Secure restraint in a stanchion or headlock is essential, as manipulation of the ear canal exposes handlers to risks such as impact from the animal’s abrupt head movements or butting. In requiring particular caution, procedures involving the bovine ear canal are similar to those given clinical consideration in human external ear disease. Unlike hair or saliva collection, which can be done quickly, earwax sampling is slower, and is more labour-intensive when undertaken using ear scoops, curettes, cotton swabs, paper towels or other small instruments. Recent advances in portable endoscopic ear-cleaning devices, equipped with replaceable ear scoops, LED lights, a micro camera and WIFI/smartphone connectivity, now enable safer visual inspection and more efficient earwax collection directly from the ear canal (16). Future studies should establish reference ranges across no-stress, cold-stress and heat-stress groups with balanced sex distribution to improve ECC reliability as a biomarker under practical farming conditions.

## Conclusion

Earwax is a practical and minimally invasive matrix for assessing chronic stress in cattle, as it gradually accumulates cortisol over time. Compared with sampling of conventional matrices such as blood or saliva, earwax collection requires fewer samplings and causes less stress to the animal. The findings indicate that sex, age and environmental factors should be considered when interpreting ECC. Standardised sampling procedures and further comparative studies using multiple matrices (*e.g.* blood, hair and saliva) are recommended to validate the use of ECC as a reliable indicator of chronic stress in bovines.
